# Functionalized SBA-15 as a protective template for CsPbBr_3_ perovskite quantum dots

**DOI:** 10.1039/d5na00868a

**Published:** 2026-01-26

**Authors:** M. P. Athira, Suja Haridas

**Affiliations:** a Department of Applied Chemistry, Cochin University of Science and Technology Kochi Kerala India sujaharidas@cusat.ac.in; b Inter-University Centre for Nanomaterials and Devices, Cochin University of Science and Technology Kochi Kerala India

## Abstract

All-metal halide perovskite quantum dots (QDs), such as CsPbBr_3_ (CPB), exhibit outstanding optoelectronic properties, but their poor thermal stability limits practical applications. In this work, CPB QDs were embedded into mesoporous SBA-15 silica matrices functionalized with amino (NH_2_) and sulfonic acid (SO_3_H) groups to enhance stability. The SBA-15 host, synthesized hydrothermally and post-functionalized, offers high thermal and chemical resilience. CPB QDs prepared *via* hot-injection retained their optical features upon incorporation, with consistent photoluminescence and absorption spectra. Structural analysis confirmed uniform QD loading (∼17 nm) within the pores. Among all supports, SO_3_H-functionalized SBA-15 provided the greatest stability improvement, attributed to strong interactions with surface ligands. This approach presents a viable pathway for stabilizing perovskite QDs in optoelectronic applications.

## Abstract 2

Schematic representation showing the weak and strong interaction of CPB with SBA-15 NH_2_ and SBA-15 SO_3_H respectively.

## Graphical abstract

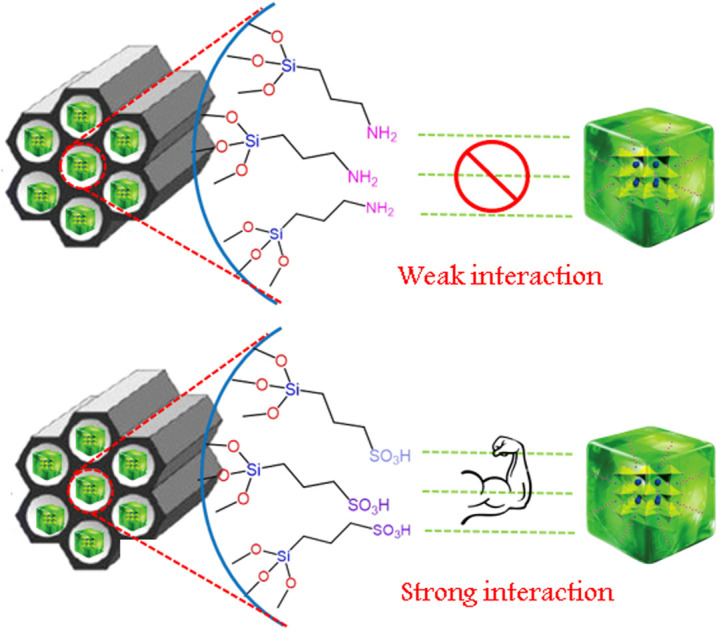

## Introduction

In recent times, there has been a growing interest in inorganic halide perovskite quantum dots (QDs), particularly CsPbX_3_ (X = Cl, Br, I).^1,2^ These QDs have garnered attention for their potential applications in light-emitting diodes (LEDs),^3^ solar cells,^4^ and lasers,^5^ owing to their superior optical properties. Compared to cadmium chalcogenide QDs,^6^ CsPbX_3_ QDs exhibit narrower emission, higher photoluminescence quantum yield (PLQY), and a tunable emission wavelength spanning the visible region. These unique emission characteristics make them especially promising as next-generation fluorescent materials for high-quality full-color displays and lighting applications.^7^ Metal halide perovskite quantum dots, being zero-dimensional nanomaterials, offer additional advantages such as a tunable emission spectrum, high light stability, and a prolonged fluorescence lifetime, attributed to the quantum confinement effect.^8^ Lead halide perovskite nanocrystals, in particular, are notable for their potential in solid-state lighting and high-definition display applications, due to their broad color gamut, high color purity, and relatively straight forward synthesis methods.

However, despite the exceptional optical performance of CsPbBr_3_ (abbreviated as CPB) QDs, a critical challenge lies in their inherent instability. Specifically, the instability of CPB quantum dots is attributed to proton transfer between the oleic acid and oleylamine ligands used during synthesis.^9^ This proton exchange leads to the loss of ligands, which destabilizes the nanocrystals and degrades their PL efficiency over time. Additionally, these QDs are sensitive to environmental factors such as moisture, oxygen, heat, and light, which further contribute to their degradation. To mitigate these stability issues, researchers have employed several techniques.^10^ One approach is encapsulating the QDs in protective matrices, such as polymers,^11^ silica^12^ or mesoporous materials,^13^ to shield them from external environmental factors. Surface passivation, using organic ligands or inorganic shell coatings, has been another strategy to reduce surface defects and improve stability by preventing ligand loss.^14^ Furthermore, chemical modifications, such as introducing functional groups or replacing unstable components like halides, are also used to enhance the QDs' resilience.^15^ These methods aim to maintain the high PLQY and narrow emission spectrum of CPB QDs by addressing their stability challenges, ultimately advancing their practical applications in optoelectronic devices.

As part of ongoing efforts to address the stability challenges of perovskite QDs, researchers have introduced various strategies, including the use of mesoporous silicon materials as templates to enhance the structural integrity of QDs.^16^ Among these, SBA-15, a mesoporous silica named after the University of California, Santa Barbara, has shown considerable promise due to its fine and controllable pore diameters, thicker walls, high surface area, and ease of synthesis and functionalization. In earlier studies, SBA-15 has been widely used as a template for incorporating a range of materials, such as metals,^17^ metal oxides,^18^ enzymes,^19,20^*etc.*, to enhance their stability/reactivity. This versatility in accommodating various materials makes SBA-15 a popular choice in fields such as catalysis, drug delivery, and energy storage. When applied to perovskite QDs, SBA-15 offers additional advantages due to its excellent thermal and mechanical stability, as well as its robust chemical resistance, which helps protect the sensitive QDs from environmental factors like heat, moisture, and oxygen that could otherwise cause rapid degradation.^21^ These properties make SBA-15 an ideal scaffold for stabilizing QDs, as it can maintain its structural integrity under challenging conditions, thus prolonging the functional lifespan of the embedded materials.

## Result and discussions

### Material characterization

Using the conventional hot-injection method (see SI for details), we synthesized CPB, SBA-15, and NH_2_- and SO_3_H-functionalized SBA-15, followed by comprehensive structural characterization.^22–26^ The morphology and particle dimensions of pristine CsPbBr_3_ (CPB) nanocrystals were examined by TEM (Fig. S1a–c). The low- and medium-magnification images [(a) and (b)] show uniformly dispersed, cubic-shaped CPB quantum dots. The corresponding particle-size-distribution histogram obtained from ImageJ analysis (Fig. S1c) confirms an average particle diameter of 17 ± 3 nm, determined from measurements of 50 individual particles. This narrow size dispersion indicates good control over nucleation and growth, resulting in uniform nanocubes. This combined evidence verifies the uniform nanoscale size and morphology of the synthesized CPB QDs, providing a reliable structural reference for their subsequent incorporation into the SBA-15 systems.^1,3,13^ To validate the formation of CPB, the absorption and emission spectra are analyzed in addition to the TEM results, as discussed in the following sections.

The SEM images (Fig. S2) confirm that the surface morphology is almost retained across all samples, indicating that the structural integrity of SBA-15 remains unaffected by functionalization. For comparison, the SEM and EDS analyses of pristine SBA-15 synthesized by the same method were recharacterized in this work (Fig. S2a–d) and found to be consistent with our earlier report.^22^ Since SEM is a surface analysis technique, the elemental composition measured reflects only the surface layer, and bulk composition may vary. However, the EDX results (Fig. S2i and S2n) provide concrete confirmation of the presence of nitrogen in SBA-15 NH_2_ and sulfur in SBA-15 SO_3_H, confirming successful functionalization. Elemental mapping images (Fig. S2f–S2h and S2k–S2m) further support this by visualizing the spatial distribution of these elements/functional groups on the surface of SBA-15.^23,27^

**Fig. 1:**
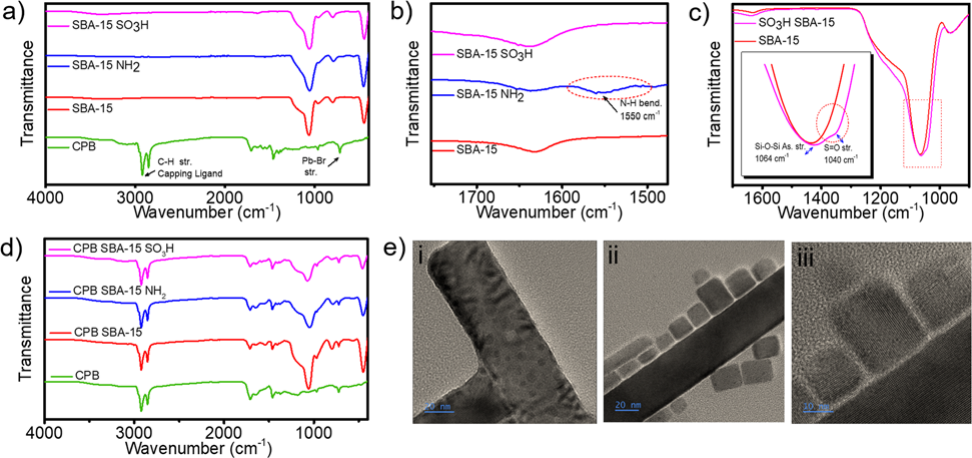
(a–d) FTIR spectra of (a) CPB, SBA-15, SBA-15-NH_2_, and SBA-15-SO_3_H showing characteristic vibrations confirming surface functionalization; (b) magnified region highlighting the N–H bending vibration at 1550 cm^−1^; (c) magnified S=O stretching region for SBA-15-SO_3_H (inset shows enlarged view of the overlapping Si–O–Si and S=O vibrations); (d) FTIR spectra of CPB, CPB@SBA-15, CPB@SBA-15-NH_2_, and CPB@SBA-15-SO_3_H demonstrating the retention of CPB features after incorporation; (e) high-resolution TEM images of CPB@SBA-15-SO_3_H showing nanocubic CsPbBr_3_ domains in close contact with the mesoporous SBA-15 framework. Scale bars: 20 nm (i, ii), 10 nm (iii).

Furthermore, we analyzed the FT-IR spectra (Fig. 1a) to confirm the successful formation of CPB and functionalization of SBA-15. The FT-IR spectrum of CPB displays distinct long-chain –(CH_2_)_*n*_– vibrations around 720 cm^−1^, symmetric and antisymmetric vibrations of COO− at 1405 and 1529 cm^−1^, and C–H stretching vibrations near 2950 cm^−1^, all attributed to the capping ligand of CPB. These peaks are clear indicators of CPB formation.^28^ For SBA-15 and its functionalized forms, a broad O–H stretching vibration is observed across 3000–3500 cm^−1^, which is attributed to the silanol (Si–OH) groups present in all SBA-15 variants. The presence of this broad O–H peak from silanol groups complicates the detection of specific N–H, O–H stretching from either NH_2_ or SO_3_H groups. Nevertheless, successful NH_2_ functionalization is confirmed in SBA-15 NH_2_ by the appearance of an N–H bending vibration at 1550 cm^−1^, which is not present in SBA-15 or SBA-15 SO_3_H, thereby distinguishing the amine-grafted sample from the other two systems (Fig. 1b). However, the presence of the SO_3_H group is confirmed by the characteristic S=O stretching vibration around 1040 cm^−1^, which is absent in SBA-15 and SBA-15 NH_2_. This region partially overlaps with the broad Si–O–Si asymmetric stretching band of the silica framework, but the SO_3_H functionality can still be distinguished by a weak shoulder near 1035–1045 cm^−1^ corresponding to the S=O stretching vibration. Such partial overlap between the S=O and Si–O–Si bands has been widely reported for sulfonic-acid-functionalized SBA-15 materials.^29,30^ This S=O peak, shown in Fig. 1c, although close to the Si–O–Si asymmetric and symmetric stretching vibrations, is unique to the SBA-15 SO_3_H spectrum, thus implying the successful functionalization with SO_3_H, consistent with earlier reports on sulfonic acid-modified mesoporous silica.^31^ These spectral features collectively validate the selective functionalization of SBA-15 and the formation of CPB.^23,27,30^

In Fig. 1d, we present the FT-IR spectral comparison of pure CPB with CPB-incorporated SBA-15 and its functionalized forms (SBA-15 NH_2_ and SBA-15 SO_3_H). The spectrum of each CPB-incorporated SBA-15 system clearly shows the major characteristic peaks of CPB, including the distinct long-chain –(CH_2_)_*n*_– vibrations around 720 cm^−1^, symmetric and antisymmetric vibrations of COO− at 1405 and 1529 cm^−1^, and C–H stretching vibrations near 2950 cm^−1^ from the capping ligand. The coexistence of characteristic CPB ligand vibrations (–(CH_2_)_*n*_–, COO^−^, and C–H stretches) with the Si–O–Si framework bands of SBA-15 suggests the successful integration of CPB within the mesoporous silica environment. Although FT-IR alone cannot conclusively confirm embedding, the preservation of these spectral features—along with complementary microscopic evidence from the CPB@SBA-15–SO_3_H composite (Fig. 1e)—supports the incorporation of CPB along or near the internal surfaces of the functionalized SBA-15 channels without disrupting its intrinsic lattice structure. This combined spectroscopic and microscopic interpretation validates the effective integration of CPB into the SO_3_H-modified SBA-15 matrix. Analogous FT-IR signatures observed for SBA-15–NH_2_ and unmodified SBA-15 suggest similar incorporation behavior across the series. Such interfacial anchoring phenomena have also been reported for CsPbBr_3_–silica composites, where surface hydroxyl and sulfonic groups serve as coordination or hydrogen-bonding sites for perovskite nanocrystals, enhancing their interfacial attachment and stability.^32,33^

Further structural confirmation of CPB incorporation was obtained from high-resolution TEM (HRTEM) analysis of the CPB@SBA-15–SO_3_H composite (Fig. 1e(i–iii)). In Fig. 1e(i), distinct dark nanocubic CsPbBr_3_ domains can be observed partially confined along or near the mesochannel walls of SBA-15–SO_3_H, indicating strong interfacial attachment of CPB. The medium-magnification image (Fig. 1e(ii)) displays additional CPB nanoparticles attached externally at the pore openings and outer surface of the silica framework, indicating that a fraction of the nanocrystals preferentially nucleated near accessible pore entrances. The lattice-resolved image (Fig. 1e(iii)) reveals well-defined lattice fringes characteristic of crystalline CsPbBr_3_, observed in direct contact with the amorphous silica wall, confirming intimate interfacial attachment between the perovskite and the SO_3_H-functionalized surface. Considering that the typical pore diameter of SBA-15 (5–10 nm) is smaller than the average CPB particle size (≈16–18 nm), complete encapsulation is unlikely. Instead, the observed morphology indicates partial confinement within the channels and strong surface anchoring at the pore boundaries. Such interfacial confinement provides effective physical protection by limiting ligand desorption and surface degradation, thereby explaining the enhanced stability of the CPB@SBA-15–SO_3_H composite. Similar confinement-mediated stabilization has been demonstrated for perovskite–silica hybrid systems.^26,32^

The preservation of open mesoporous channels in all composites indicates that pore blockage during perovskite loading is minimal. Although N_2_ adsorption–desorption (BET) measurements were not performed in the present work, previous studies on CsPbBr_3_–SBA-15 hybrids prepared under comparable conditions have reported negligible (<10%) decreases in specific surface area and pore volume after QD incorporation.^30,31^ These findings suggest that the *in situ* impregnation procedure employed here likely maintains the intrinsic textural properties of SBA-15 while enabling uniform interfacial anchoring of the perovskite nanocrystals.

### Photoluminescence studies

The absorption and PL spectra of the freshly synthesized CPB and CPB impregnated onto SBA-15, SBA-15 NH_2_, and SBA-15 SO_3_H were examined and displayed in Fig. S3. The absorption and emission spectra of both functionalized and bare SBA-15-impregnated CPBs exhibited the same trend as that of bare CPB, indicating that the mesoporous silica-supported perovskite nanoparticles did not induce any changes in the absorption/emission characteristics of CPB. Essentially, the absorption parameters remained consistent upon supporting CPB with mesoporous silica. Furthermore, the PL spectra recorded at an excitation wavelength of 365 nm, retained the characteristic CPB emission maxima near 514 nm.

The average pore diameter of SBA-15 systems (typically 5–10 nm, occasionally extending up to 20–30 nm depending on synthesis conditions) provides a favorable nanoscale environment for perovskite loading. Although the pristine CPB particles synthesized *ex situ* exhibit an average size of approximately 17 nm, the *in situ* formation of CPB within the mesoporous SBA-15 channels can lead to self-limited growth of smaller nanocrystals due to geometric confinement. This size-adjustment mechanism, governed by the pore walls, enables partial accommodation of CPB nanocrystals near the mesopore openings and along the internal channels. Consequently, interfacial anchoring and spatial confinement within SBA-15 are expected to enhance the structural and environmental stability of the CPB phase, even without complete encapsulation inside every pore.^9,26^ Similar interfacial confinement-driven stabilization mechanisms have recently been discussed for hybrid perovskite nanostructures embedded in porous oxide matrices. This interpretation aligns with the HRTEM evidence, where partial confinement and surface anchoring of CPB nanocrystals were directly observed. Liu *et al.* reported, in 2021,^26^ that impregnation into non-functionalized SBA-15 enhances the thermal stability of CPB. The stability of CPB is directly affected by the interaction between perovskite and the associated capping ligands (oleic acid and oleyl amine).

In our study, alongside examining bare SBA-15, the competency of the NH_2_- and SO_3_H-functionalized SBA-15 is also scrutinized. This approach aims to achieve a stable perovskite, harnessing the interaction of functional groups NH_2_ and SO_3_H with the capping ligands and perovskites.^33^

**Fig. 2:**
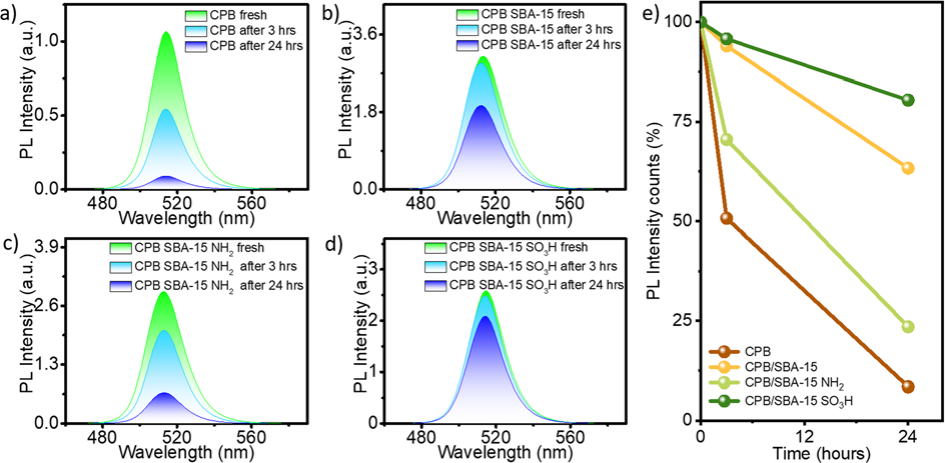
Time dependent PL studies of the systems (a) CPB, (b) CPB SBA-15, (c) CPB SBA-15 NH_2_, and (d) CPB SBA-15 SO_3_H. (e) Comparison of time dependent PL variation among the composites (PL intensity represented in %).

The stability of perovskite quantum dots can be easily gauged through monitoring of the reduction in PL intensity. PL spectra of as-synthesized CPB were recorded after dispersion in toluene. It was observed that nearly 50% of the emission diminished in 3 hours of toluene dispersion (Fig. 2a). Interaction of toluene with the capping ligand causes deformation in the perovskite structure.^33^ SBA-15 integrated CPBs also displayed a lowering of emission intensity with time; the extent of deviation being dependent on the functionality. Specifically, the CPB incorporated into bare SBA-15 experienced only a 6% quenching of emission after 3 hours of toluene dispersion, maintaining 63% emission even after 24 hours (Fig. 2b). Among NH_2_ and SO_3_H functionalized SBA-15 (Fig. 2c and d), the SO_3_H variant demonstrates remarkable stability to CPB, with less than 5% emission intensity quenched after 3 hours. This system sustains over 80% of its emissions even after 24 hours. In contrast, the NH_2_ functionalized system provides comparatively lower stability for CPB, even though a slight enhancement in stability relative to bare SBA-15 was evident. A comparative evaluation of the emission intensity with different samples is provided in Fig. 2e. The –SO_3_H functional group within porous SBA-15 likely interacts with the capping ligands of perovskite nanomaterials, enhancing its longevity, as previously reported for similar functionalized mesoporous supports.^26^

**Fig. 3:**
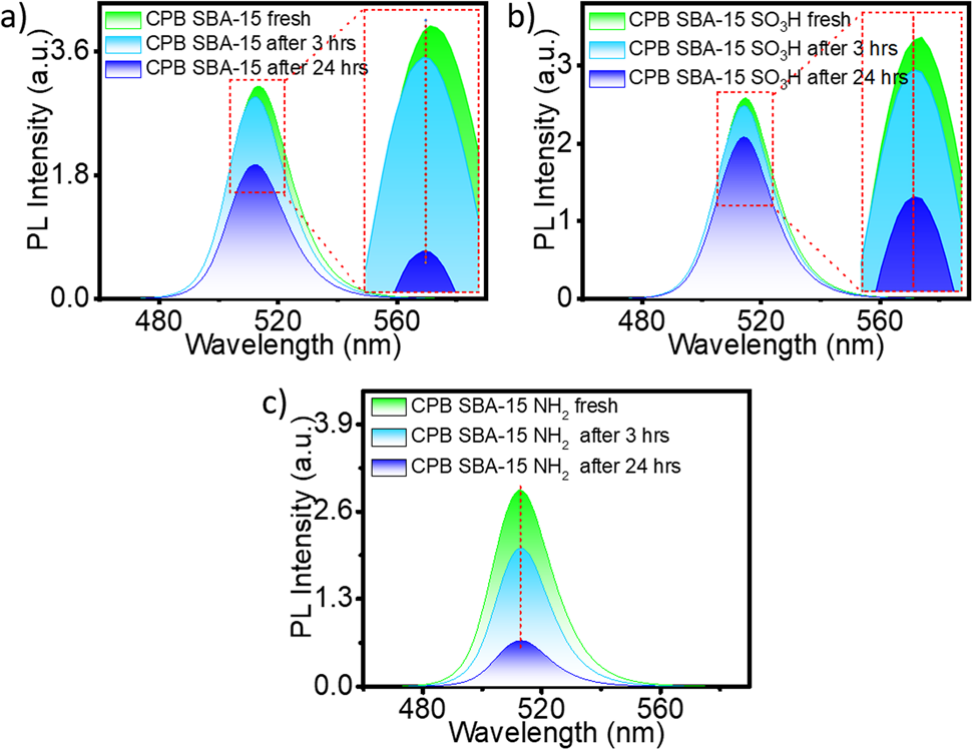
Time-dependent PL intensity variation of (a) CPB @ SBA-15, (b) CPB @ SBA-15-SO_3_H, and (c) CPB @ SBA-15-NH_2_ composites recorded under identical conditions. Insets show minor blue shifts in emission maxima for the SBA-15 and SBA-15-SO_3_H systems. (Each data set was verified for consistency across repeated measurements from the same batch, confirming the reproducibility of the observed PL stability trends).

A slight blue shift (∼1–2 nm) in PL emission was observed during quenching for both SBA-15 and the SBA-15–SO_3_H templates (Fig. 3a and b), but not for SBA-15–NH_2_ (Fig. 3c). The PL intensity decay measurements were repeated on multiple aliquots from the same batch to verify consistency, and the data sets showed negligible variation, confirming the reliability of the observed emission stability trends. The enhanced PL stability is plausibly related to the synergistic effects of both the mesoporous SBA-15 framework and the attached functional groups, particularly –SO_3_H. The confined channels of SBA-15 create an inert environment that shields the perovskite nanocrystals from moisture and oxygen, while the acidic –SO_3_H groups can engage in weak hydrogen-bonding or electrostatic interactions with the capping ligands of CsPbBr_3_. These interactions likely suppress ligand detachment and slow degradation processes, thereby preserving luminescence intensity over extended durations. Such interfacial stabilization mechanisms have been reported for sulfonic-acid-modified silica systems. Nevertheless, further spectroscopic and thermal analyses are required to elucidate the detailed nature of these interactions.

To further clarify the nature of the SO_3_H–CPB interaction, the stabilization mechanism is attributed primarily to hydrogen-bonding and electrostatic associations rather than direct coordination. The acidic –SO_3_H groups can engage in hydrogen bonding or ionic pairing with the amine and carboxylate termini of the oleylamine/oleic-acid capping ligands present on the CPB surface. No additional vibrational bands or major peak shifts appear in the FT-IR spectra that would signify new covalent or coordination-type bonds. Likewise, the preservation of the CPB lattice fringes in HRTEM and the unaltered XRD reflections confirm that the perovskite crystal structure remains intact. These observations support a non-covalent interfacial attachment mechanism consistent with previous reports on perovskite–silica hybrid systems, where surface acid–base and hydrogen-bonding interactions dominate the stabilization process.^26,32,33^

**Scheme 1:**
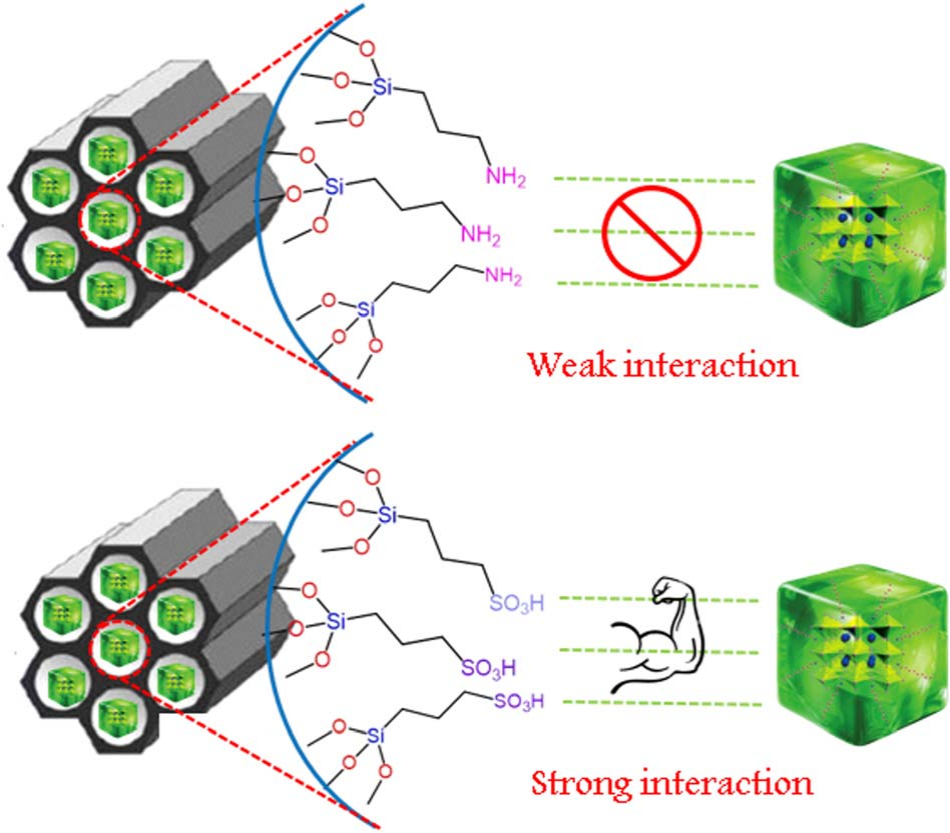
Schematic illustration showing the probable interfacial interactions between CPB and functionalized SBA-15 templates. The interaction of CPB with SBA-15 SO_3_H is depicted as stronger relative to SBA-15 NH_2_, based on the comparative PL stability trends. The schematic is qualitative and intended only to visualize possible hydrogen-bonding or electrostatic associations inferred from experimental behavior.

The PL shift is likely due to minor electronic interactions between CPB and the SO_3_H groups.^29^ The acidic nature of SO_3_H groups may withdraw some electron density from CPB, subtly altering its electronic environment. Consequently, this interaction leads to a blue shift, indicating a higher energy emission, which suggests an interaction between CPB and the acidic SO_3_H groups of the SBA-15 template. A schematic representation of the weak interaction of CPB with NH_2_ functionalized SBA-15 and a strong interaction with SO_3_H functionalized SBA-15 is schematically represented (Scheme 1).

Similar qualitative distinctions between hydrogen-bonding and acid–base interfacial interactions influencing the stabilization of perovskite nanocrystals within functionalized silica matrices have been reported in literature.^26,32^ These reports support the proposed interpretation that the –SO_3_H functionality can form stronger interfacial associations with CPB surface ligands than –NH_2_, contributing to the enhanced PL stability observed.

## Conclusions

We report the successful synthesis and characterization of perovskite quantum dot (CPB) composites supported on bare and functionalized SBA-15 templates. Incorporation into the mesoporous silica framework substantially enhances the structural and photoluminescence stability of CPB. Among the studied systems, CPB@SBA-15–NH_2_ exhibits a moderate improvement in stability relative to bare SBA-15, likely due to partial surface passivation through amine–ligand interactions. In contrast, CPB@SBA-15–SO_3_H displays the most pronounced stabilization, maintaining over 80% of its emission after 24 hours of toluene dispersion. This superior performance is attributed to stronger interfacial associations—hydrogen-bonding and electrostatic—between the acidic SO_3_H groups and the surface ligands of the perovskite nanocrystals. Overall, the comparative study highlights the decisive role of surface functional chemistry in controlling perovskite–matrix interactions, demonstrating that SO_3_H-modified SBA-15 provides a robust route toward highly stable, mesoporous silica-supported perovskite nanocomposites.

## Author contributions

Athira M. P.: visualization, methodology, data curation, conceptualization, investigation, writing – original draft. Suja Haridas: validation, methodology, supervision, conceptualization, manuscript correction.

## Conflicts of interest

There are no conflicts to declare.

## Supplementary Material

NA-008-D5NA00868A-s001

## Data Availability

The data supporting this article have been included as part of the supplementary information (SI). Supplementary information: experimental details, morphological characterization (SEM and TEM images) and photoluminescence studies. See DOI: https://doi.org/10.1039/d5na00868a.

## References

[cit1] Protesescu L., Yakunin S., Bodnarchuk M. I., Krieg F., Caputo R., Hendon C. H., Yang R. X., Walsh A., Kovalenko M. V. (2015). Nano Lett..

[cit2] Clinckemalie L., Valli D., Roeffaers M. B. J., Hofkens J., Pradhan B., Debroye E. (2021). ACS Energy Lett..

[cit3] Pan Y., Zhang Y., Kang W., Deng N., Yan Z., Sun W., Kang X., Ni J. (2022). Mater. Adv..

[cit4] Zhang J., Hodes G., Jin Z., Liu S. (2019). Angew. Chem., Int. Ed..

[cit5] Wang X., Shoaib M., Wang X., Zhang X., He M., Luo Z., Zheng W., Li H., Yang T., Zhu X., Ma L., Pan A. (2018). ACS Nano.

[cit6] Xiang X., Wang L., Zhang J., Cheng B., Yu J., Macyk W. (2022). Adv. Photonics Res..

[cit7] Ma T., Wang S., Zhang Y., Zhang K., Yi L. (2020). J. Mater. Sci..

[cit8] Yang D., Huo D. (2020). J. Mater. Chem. C.

[cit9] Cai Y., Wang L., Zhou T., Zheng P., Li Y., Xie R.-J. (2018). Nanoscale.

[cit10] Liu J., Yang Z., Ye B., Zhao Z., Ruan Y., Guo T., Yu X., Chen G., Xu S. (2019). J. Mater. Chem. C.

[cit11] Raja S. N., Bekenstein Y., Koc M. A., Fischer S., Zhang D., Lin L., Ritchie R. O., Yang P., Alivisatos A. P. (2016). ACS Appl. Mater. Interfaces.

[cit12] R S., Nayak V., Jyothi M. S., Geetha Balakrishna R. (2020). J. Alloys Compd..

[cit13] Gao F., Yang W., Liu X., Li Y., Liu W., Xu H., Liu Y. (2021). Chem. Eng. J..

[cit14] Cao Y., Zhu W., Li L., Zhang Z., Chen Z., Lin Y., Zhu J.-J. (2020). Nanoscale.

[cit15] Ding Y., He B., Zhu J., Zhang W., Su G., Duan J., Zhao Y., Chen H., Tang Q. (2019). ACS Sustain. Chem. Eng..

[cit16] Carulli F., He M., Cova F., Erroi A., Li L., Brovelli S. (2023). ACS Energy Lett..

[cit17] Boutros M., Trichard J.-M., Da Costa P. (2009). Appl. Catal., B.

[cit18] Singh S., Kumar R., Setiabudi H. D., Nanda S., Vo D.-V. N. (2018). Appl. Catal., A.

[cit19] Salvi H. M., Yadav G. D. (2019). Biocatal. Agric. Biotechnol..

[cit20] Takimoto A., Shiomi T., Ino K., Tsunoda T., Kawai A., Mizukami F., Sakaguchi K. (2008). Microporous Mesoporous Mater..

[cit21] Kundu S., Kelly T. L. (2020). EcoMat.

[cit22] Athira M. P., Arun R., Haridas S. (2024). J. Porous Mater..

[cit23] Parambadath S., Mathew A., Barnabas M. J., Kim S. Y., Ha C.-S. (2016). J. Sol-Gel Sci. Technol..

[cit24] Arun R., Athira M. P., Remello S. N., Haridas S. (2023). React. Kinet. Mech. Catal..

[cit25] Vighnesh K., Wang S., Liu H., Rogach A. L. (2022). ACS Nano.

[cit26] Chen H., Wang Y., Wang J., Liu W. (2021). Coatings.

[cit27] Gabla J. J., Lathiya D. R., Revawala A. A., Maheria K. C. (2019). Res. Chem. Intermed..

[cit28] Wang X., Zhuo S., Fu J., Li X., Zhao X., Jiang H., Lv G., Li P., Li J., Zhang W.-H., Ma W. (2023). ACS Appl. Mater. Interfaces.

[cit29] Shi J., Ge W., Zhu J., Saruyama M., Teranishi T. (2020). ACS Appl. Nano Mater..

[cit30] Ganji S., Mutyala S., Neeli C. K. P., Rao K. S. R., Burri D. R. (2013). RSC Adv..

[cit31] Pečar D., Goršek A. (2019). React. Kinet. Mech. Catal..

[cit32] Collantes C., Teixeira W., González-Pedro V., Bañuls M.-J., Quintero-Campos P., Morais S., Maquieira Á. (2023). Dalton Trans..

[cit33] Fiuza-Maneiro N., Sun K., López-Fernández I., Gómez-Graña S., Müller-Buschbaum P., Polavarapu L. (2023). ACS Energy Lett..

