# Supplementary material for: Functionalized SBA-15 as a protective template for CsPbBr3 perovskite quantum dots
Source: Nanoscale Adv. 2026 Jan 26;8(3):829–34. doi: 10.1039/d5na00868a (PMC12833752; doi:10.1039/d5na00868a)
Supplement: NA-008-D5NA00868A-s001 [file NA-008-D5NA00868A-s001.pdf]

Supplementary Information (SI) for Nanoscale Advances.
This journal is © The Royal Society of Chemistry 2026

SUPPORTING INFORMATION

Functionalized SBA-15 as a Protective Template for $CsPbBr_3$ Perovskite Quantum Dots

Athira M P[a], Suja Haridas[a,b]
[a] *Department of Applied Chemistry, Cochin University of Science and Technology, Kochi, Kerala, India.*

[b] *Inter-University Centre for Nanomaterials and Devices, Cochin University of Science and Technology, Kochi, Kerala, India*

sujaharidas@cusat.ac.in

Materials and Methods

Chemicals

Pluronic 123 (P123), tetraethyl orthosilicate (TEOS), 3-aminopropyl-triethoxysilane (APTES), 3-mercaptopropyl-trimethoxysilane (MPTMS), cesium carbonate (Cs_2CO_3) (99.9%), lead bromide ($PbBr_2$) (99.999%), oleic acid (OA, technical grade 90%), oleylamine (OM, technical grade 70%), and octadecene (ODE, technical grade 90%) were purchased from Sigma-Aldrich. Hydrochloric acid (HCl, 35%), sulphuric acid (H_2SO_4), hydrogen peroxide (H_2O_2), ethanol, methanol and toluene were purchased from Merck. All reagents and solvents were of analytical grade and used as such without any further purification.

Synthesis of mesoporous supports

Synthesis of SBA-15: SBA-15 was prepared according to a previously reported protocol from tetra ethyl ortho silicate precursor using P123 as template [1].

Synthesis of SBA-15 NH_2: Amine functionalized SBA-15 (abbreviated as SBA-15 NH_2), 0.5g of SBA-15, 1.17 ml of APTES and 50 ml toluene were added to 100 ml round bottom flask and refluxed at 85 °C. After 24 hours the resulting mixture was filtered, washed with toluene and ethanol, and dried at room temperature followed by oven drying at 60 °C for 24 h [2]. A schematic representation of this process is provided in Scheme S1.

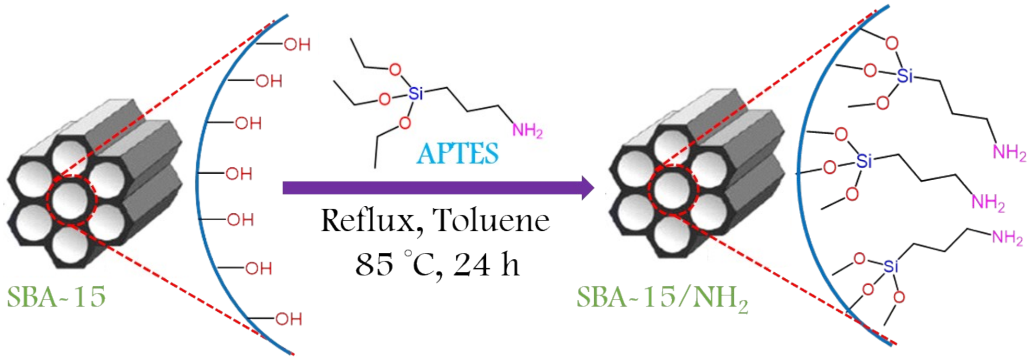

Scheme S1: Synthetic pathway for SBA-15 NH_2

*Synthesis of SBA-15 SO_3H***:** Sulphonic acid functionalized SBA-15 (abbreviated as SBA-15 SO_3H) Calcined SBA-15 was activated for 30 minutes, and the functionalization was done as follows. To 0.5 g calcined SBA-15, 5 mL MPTMS and 10 mL toluene were added and refluxed for 6 h at 60 °C. The precipitate after filtering was washed with methanol and distilled water and dried overnight at 70 °C to get thiol functionalized SBA-15. Oxidation of the thiol moiety with 5mL H_2O_2 and 1 drop H_2SO_4 followed by filtering, washing and drying gave sulphonic acid functionalized SBA-15 [3], which is denoted as SBA-15 SO_3H (depicted in Scheme S2).

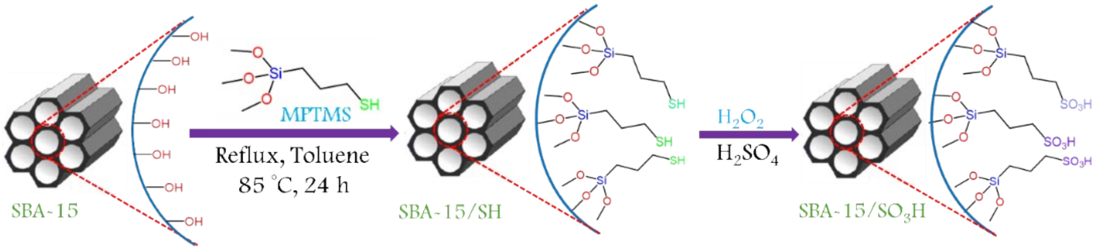

Scheme S2: Synthetic pathway for SBA-15 SO_3H

Synthesis of CPB QDs, CPB SBA-15 and CPB SBA-15 X (X= NH_2 or SO_3H):

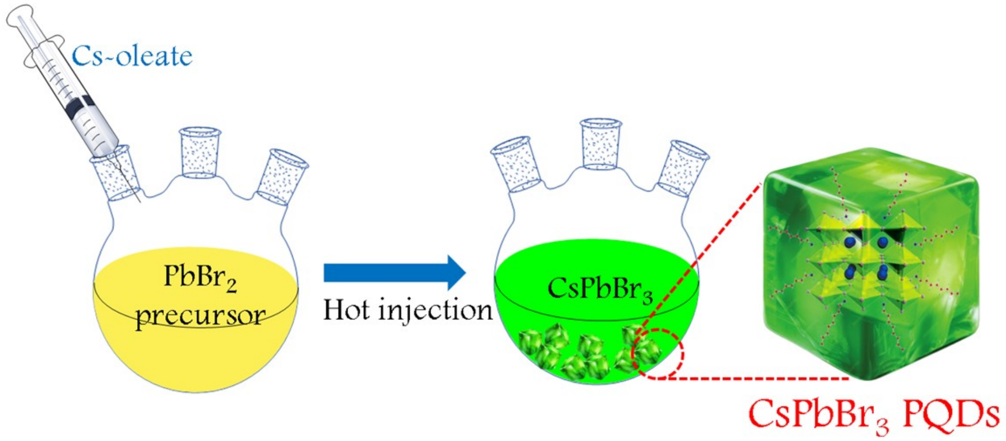

Scheme S3: Reaction pathway for CPB QDs

The synthesis of CPB perovskite quantum dots (CPB QDs) was carried out using the hot injection method, following a previously reported procedure [4]. In brief, lead bromide precursor was dissolved in a mixture of oleic acid and oleylamine, which acts both as stabilizing agent and ligand for the quantum dots. This mixture was heated under an inert atmosphere, and cesium precursor solution was rapidly injected into the reaction vessel at high temperatures, typically around 180°C. The sudden temperature change facilitates the nucleation of CPB nanocrystals. Following this, the mixture was cooled, and the nanocrystals were separated via centrifugation (shown in Scheme S3).

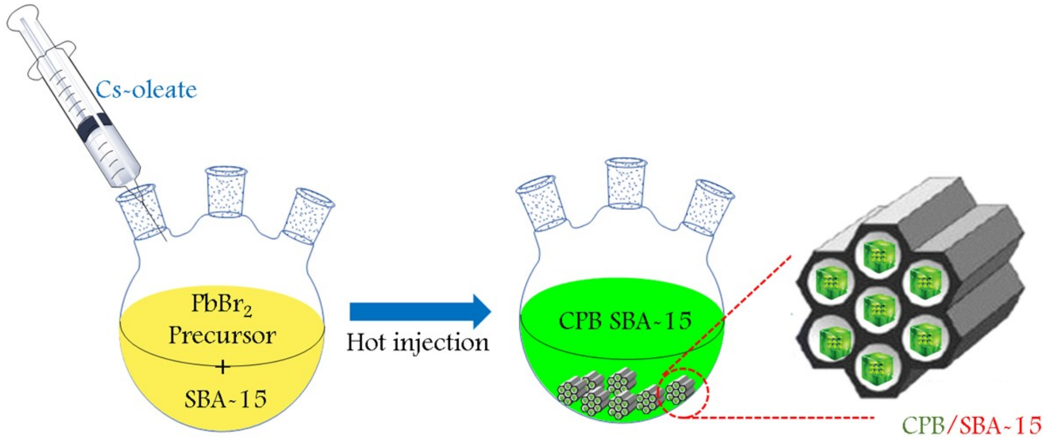

Scheme S4: Reaction pathway for modified CPB SBA-15

The SBA-15 integrated CPB (CPB SBA-15) was synthesized through a slightly modified version of the reported CPB hot injection method. Before injecting Cs-oleate into the mixture of $PbBr_2$-octadecene, SBA-15 was introduced and maintained for 30 minutes under heating conditions. This modification in the procedure aimed at in situ incorporation of CPB into the SBA-15 framework [5] imparts enhanced stability and other desirable properties to the resulting QDs (presented in Scheme S4). A similar procedure was adopted for the synthesis of CPB SBA-15 X (X= NH_2 or SO_3H), instead of bare SBA-15, the functionalized SBA-15 is utilized.

Characterization techniques

The steady state PL spectra was obtained from the Horiba Fluorolog-3 fluorescence spectrometer at an excitation wavelength of 365 nm. Functional group analyses were achieved by Fourier transform infrared spectroscopy (FTIR) spectra recorded on the JASCO model 4100 FTIR spectrometer. The scanning electron microscope-energy dispersive X-ray (SEM-EDX) data was collected using a JEOL JSM-5600 LV scanning electron microscope. The textural characterization of Perovskite QDs were studied by using FEI-TECNAI T30 high-resolution transmission electron microscopy (HR-TEM) at an accelerating voltage of 300 kV.

Results and Discussion

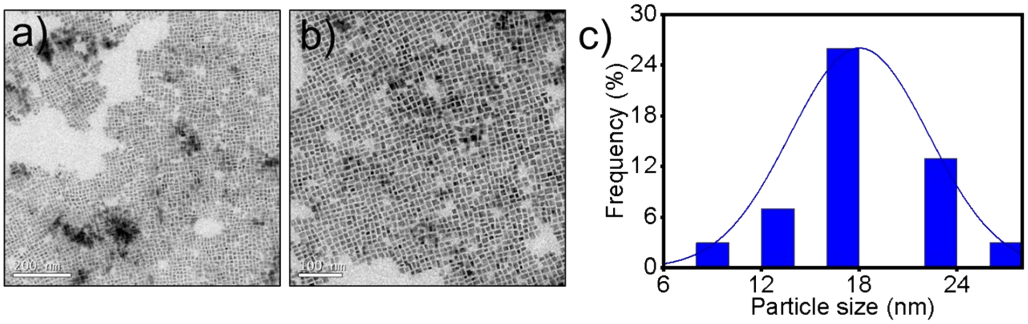

Fig. S1: TEM analysis of pristine $CsPbBr_3$ (CPB) quantum dots: (a) low-magnification image (200 nm scale), (b) medium-magnification image (100 nm scale), and (c) particle-size-distribution histogram obtained from ImageJ analysis. The X-axis represents particle diameter (nm) and the Y-axis represents frequency (%). The histogram confirms a mean particle diameter of 17 ± 3 nm, determined from measurements of 50 individual particles, indicating a narrow size distribution and uniform cubic morphology.

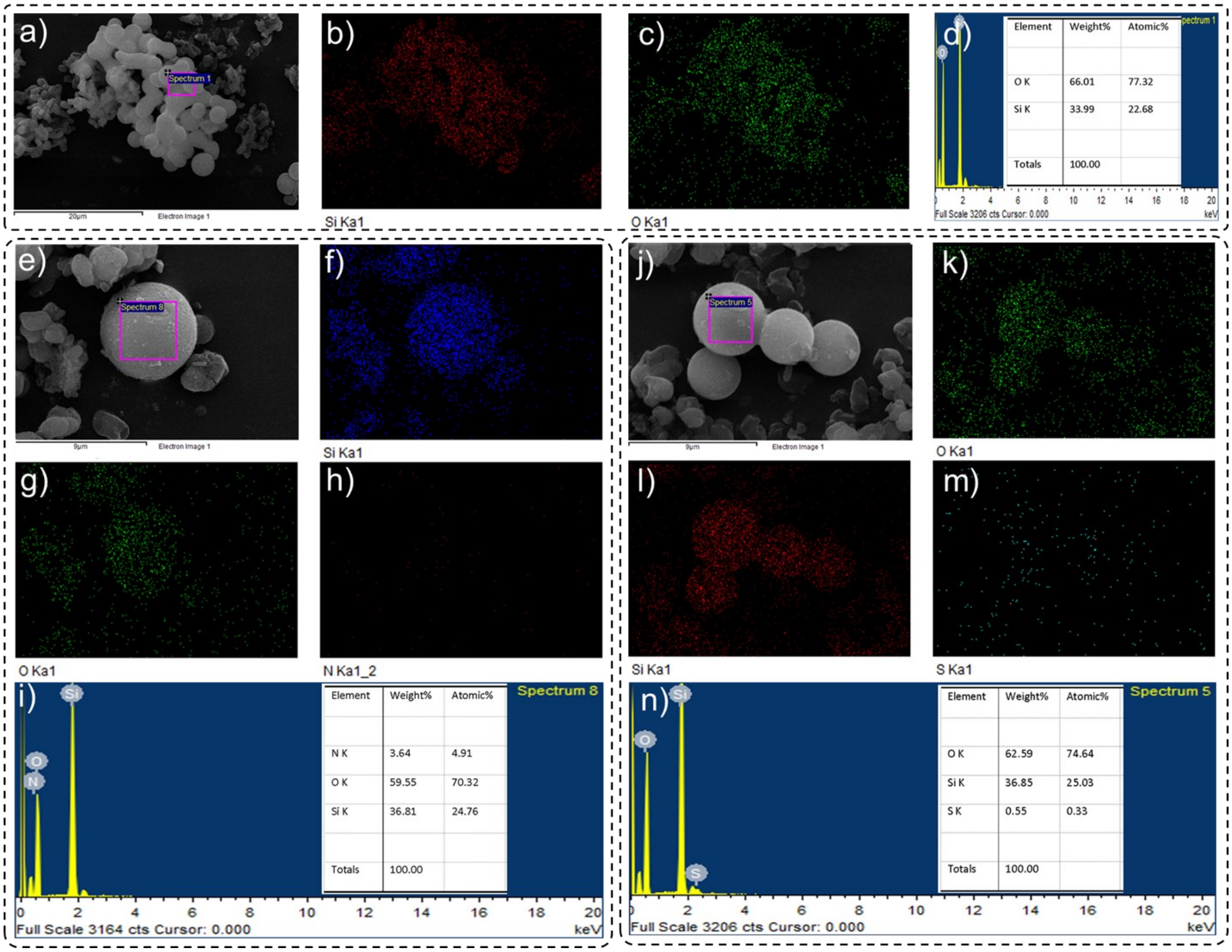

Fig. S2: SEM images, elemental mapping, and EDX spectra of (a–d) pristine SBA-15, (e–i) SBA-15–NH_2, and (j–n) SBA-15–SO_3H. The elemental maps correspond to Si, O, and N (for SBA-15–NH_2) and Si, O, and S (for SBA-15–SO_3H), confirming successful surface functionalization of the mesoporous silica. All SEM images include scale bars of 9–20 µm as indicated.

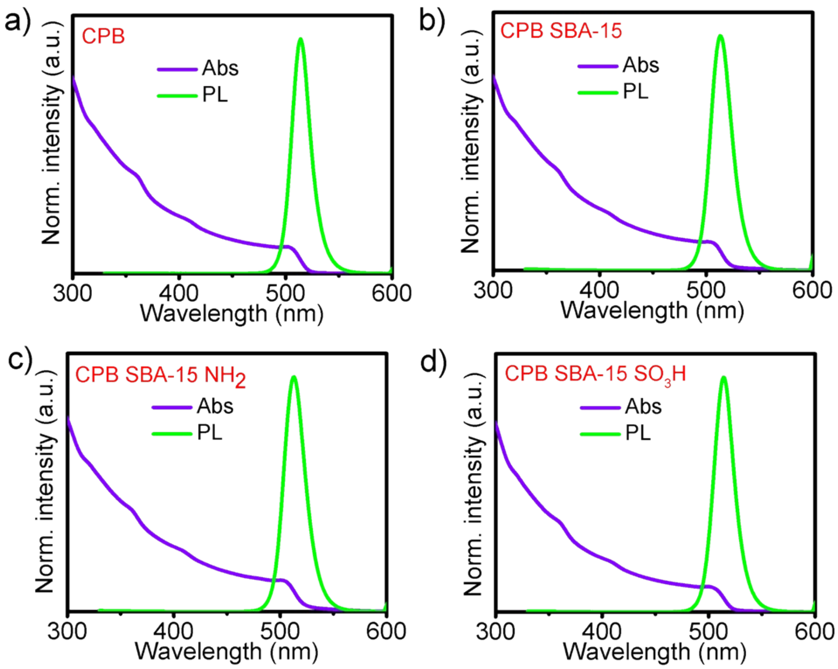

Fig. S3: Absorption and PL spectra of the as-synthesized fresh samples of CPB, CPB SBA-15, CPB SBA-15 NH_2, and CPB SBA-15 SO_3H.

References

1 M. P. Athira, R. Arun and S. Haridas*,* *J Porous Mater*, 2024, **31**, 305–316

2 S. Parambadath, A. Mathew, M. J. Barnabas, S. Y. Kim and C.-S. Ha*, J Sol-Gel Sci Technol*, 2016, **79**, 426–439.

3 R. Arun, M. P. Athira, S. N. Remello and S. Haridas, *Reac Kinet Mech Cat*, 2023, **136**, 2277–2294

4 K. Vighnesh, S. Wang, H. Liu and A. L. Rogach*, ACS Nano*, 2022, **16**, 19618–19625

5 H. Chen, Y. Wang, J. Wang and W. Liu, *Coatings*, 2021, **11**, 953.
